# Dengue and Typhoid: A Duet of Choreoathetosis

**DOI:** 10.7759/cureus.70292

**Published:** 2024-09-26

**Authors:** Harsha Vardhan Gudibandi, Harini Chinnaraj, Maddina Vinay Vardhan, J Kumar, Subramaniyan Kumarasamy

**Affiliations:** 1 General Medicine, Sri Ramaswamy Memorial (SRM) Medical College Hospital and Research Centre, SRM Institute of Science and Technology (SRMIST), Chengalpattu, IND

**Keywords:** athetosis, cerebral movement disorders, chorea-ballism, dengue virus infection, typhoid infection

## Abstract

Choreoathetoid movements can be caused by a range of conditions. Here, we discuss the case of a 16-year-old male with a history of acute febrile illness who presented with features typical of paroxysmal dystonic choreoathetosis. He arrived at the hospital complaining of a fever that had been present for four days, suggesting a viral etiology, but he did not exhibit any involuntary movements. The routine panel suggests both dengue and typhoid as potential culprits. Consequently, during the ward stay, the patient developed involuntary movements in bilateral upper limbs, lower limbs, and face. Co-infection with both dengue and typhoid is seldom seen, with an increasing number of cases in the recent few years. These situations can sometimes put the treating physician in a difficult situation with respect to management. This case is being discussed because of its rare manifestation caused by a rare co-infection.

## Introduction

Choreoathetosis is a term used to describe both chorea and athetosis due to their frequent combination in presentation. Chorea is a movement disorder that causes irregular, fluidly changing movements that are unsustained. Athetosis is defined as subtle writhing or twisting movement, which is often referred to as a slower form of chorea. The movements are typically limb-related, can be infrequently facial, and range in severity from mild, intermittent abnormalities of gesture and expression, fidgeting fingers and hands, and an unstable dance-like gait to a constant flow of violent, entirely involuntary movements [1]. Dengue fever is a virus-borne infection transmitted by the mosquito *Aedes aegypti* and is endemic in Southeast Asia among all parts of the world [2]. Although the presentation of dengue fever is obvious, atypical presentations such as myocarditis, pericarditis, transaminitis, acalculous cholecystitis, and acute respiratory distress syndrome (ARDS) are not uncommon [3]. Typhoid fever, also known as enteric fever, is a deadly disease in developing countries. It is a systemic infection caused by the human-adapted pathogen *Salmonella enterica* serovar Typhi or *Salmonella typhi* [4]. Here, we present an interesting case with co-infection of dengue virus (DV) (confirmed by serum IgM enzyme-linked immunosorbent assay (ELISA)) and typhoid encephalopathy (confirmed by blood cultures, serum IgM ELISA, and cerebrospinal fluid (CSF) reverse transcription-polymerase chain reaction (RT-PCR) for *Salmonella typhi*), manifesting choreoathetoid movements in the fever ward.

## Case presentation

A 16-year-old school student presented to the casualty with his mother with complaints of fever for the last four days. He was apparently healthy and asymptomatic until four days back, attending his school regularly, after which he had a high-grade fever with chills and rigors, which was intermittent, responding to antipyretics at home initially. He also had three episodes of vomiting for the last two days. His mother brought him to the hospital as the fever episodes were persistent. The boy reported a dull, persisting headache behind his eyes and generalized body ache with a similar onset as the fever. The boy and his mother denied any history of major or chronic illness or in-hospital admissions in the past. He has not taken any herbal (native) medication or vitamin supplements and was not abusing any psychoactive substances. He had no history of recent travel, and none of the family members were experiencing similar symptoms.

On examination, the patient was conscious and oriented to time, place, and person, and his Glasgow Coma Scale score was 15/15. He was moderately built with a body mass index (BMI) of 21 kg/m^2^, his pulse was regular at 106 beats/minute, his blood pressure was 120/70 mmHg, his temperature was 100.2°F, and his oxygen saturation was 99% without any support. His blood glucose was 122 mg/dL. His tongue appeared dry. There was no sign of rash in any form and no sign of any involuntary movements of limbs or face. There were no signs of pallor, icterus, lymphadenopathy, clubbing, cyanosis, and localized or generalized edema. He conveyed mild generalized tenderness on palpation of the abdomen without any signs of organomegaly. Examination of cardiovascular, respiratory, and central nervous systems were normal at the time.

The patient was admitted to the fever ward with an initial impression of an unspecified viral infection. He was started on symptomatic treatment while initial investigations were done, which showed as mentioned in Table 1. Dengue non-structural protein 1 (NS1) was sent in view of retro-orbital pain and demographic history and was positive, which eventually was confirmed by serum IgM for the dengue virus. Serum IgG for the same was negative.

**Table 1:** Initial blood investigation report WBC: white blood cells, SGOT: serum glutamic-oxaloacetic transaminase, SGPT: serum glutamic-pyruvic transaminase, ALP: alkaline phosphatase, GGT: gamma-glutamyl transferase

Parameters	Results	Reference
Blood		
Hemoglobin	13.9 g/dL	13-17 g/dL
WBC count	4,860/cu mm	4000-11,000/cu mm
Neutrophil	75.8%	40%-80%
Lymphocytes	20.4%	20%-40%
Eosinophil	0%	1%-4%
Basophil	0.2%	0%-2%
Monocytes	0.6%	2%-10%
Platelet	99,000/cu mm	150,000-410,000/cu mm
Renal function tests		
Urea	20 mg/dL	17-43 mg/dL
Creatinine	0.7 mg/dL	0.6-1.2 mg/dL
Electrolytes		
Sodium	134 mmol/L	135-145 mmol/L
Potassium	3.8 mmol/L	3.5-5.5 mmol/L
Chloride	99 mmol/L	98-107 mmol/L
Bicarbonate	27 mmol/L	21-31 mmol/L
Liver function tests		
Total bilirubin	1.61 mg/dL	0.5-1 mg/dL
Direct bilirubin	0.84 mg/dL	Unto 0.3 mg/dL
SGOT	265 U/L	<35 U/L
SGPT	92 U/L	<45 U/L
ALP	231 U/L	52-171 U/L
GGT	289 IU/L	<55 IU/L
Albumin	3 g/dL	3.5-5.2 g/dL
Globulin	3 g/dL	2.5-3 g/dL
Total protein	6 g/dL	5.7-8 g/dL

**Figure 1:**
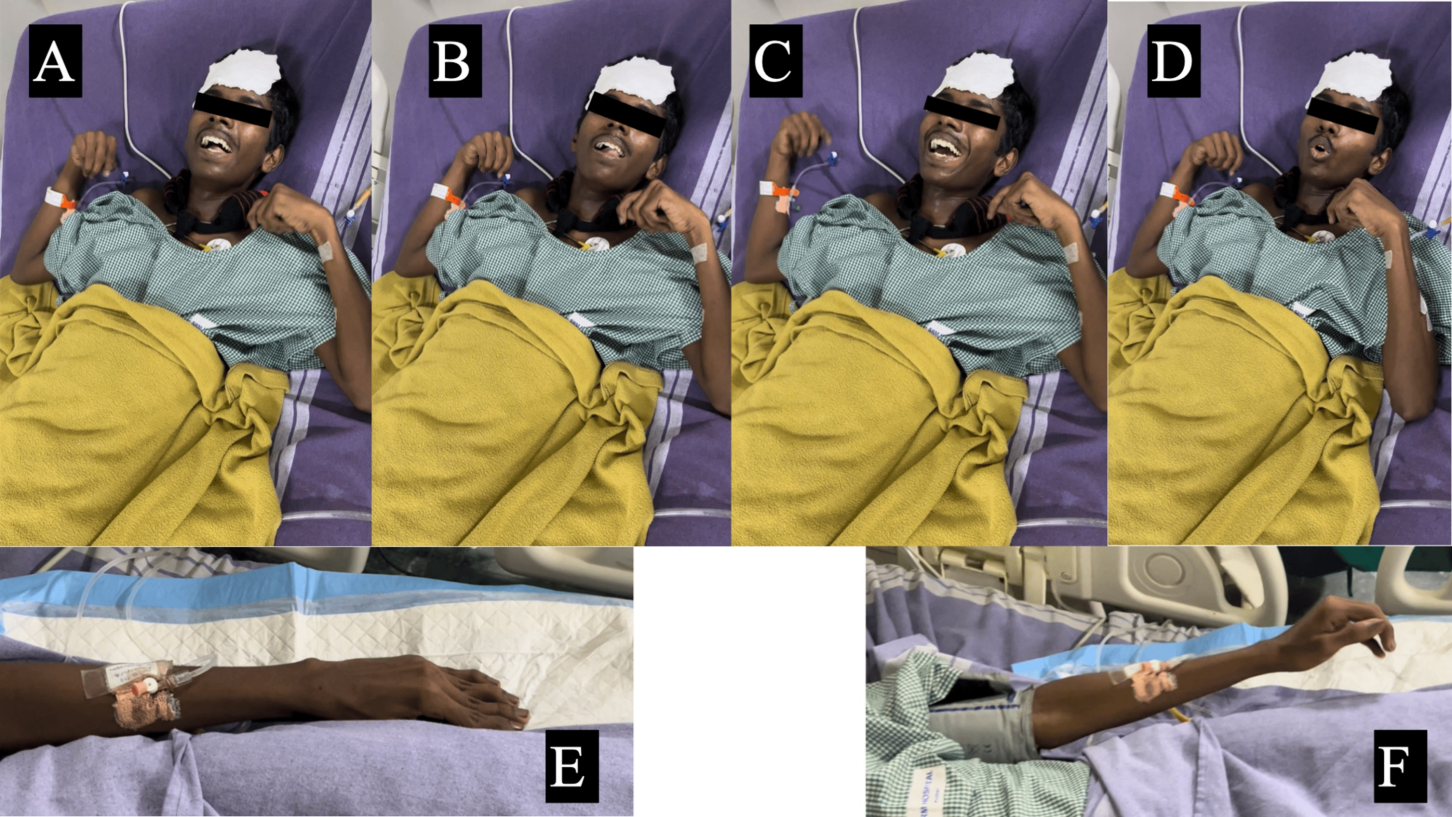
Collection of images showing abnormal movements in the face and upper limbs A, B, C, and D: Abnormal movements in the face and bilateral upper limbs. E and F: Abnormal movements in the left upper arm.

After two days of in-hospital stay, the patient developed involuntary movements of all four limbs, which were asymmetrical and predominantly seen in the upper limbs. The movements were rapid, alternating in frequency with occasional twisting movements of the left upper limb. He also developed a high-grade fever of 102.4°F after 36 hours of afebrile period. The involuntary movements were rapidly progressive with increasing amplitude, involving the face with rapid blinking and motor tics (Figure 1). He was conscious but had confused orientation throughout the time of involuntary movements. The movements were partly suppressed with an intravenous benzodiazepine for some time but have not subsided completely. Blood cultures were sent to look for other infections.

The patient was shifted to an intensive care ward and subsequently intubated in view of a low Glasgow Coma Scale score. Cerebrospinal fluid analysis was done and showed increased protein and mild lymphocytosis (Table 2).

**Table 2:** Cerebrospinal fluid analysis report CSF: cerebrospinal fluid, RBCs: red blood cells, LDH: lactate dehydrogenase, RT-PCR: reverse transcription-polymerase chain reaction

CSF analysis	Results	Reference range
Opening pressure	10 cm H2O	8-18 cm H2O
Appearance	Clear	Clear
Cells	7-8 cells (lymphocytes), no RBCs	<5 cells
Proteins	49 mg/dL	<45 mg/dL
Glucose	68 mg/dL	50-75 mg/dL
LDH	35 U/L	<40 U/L
Chloride	120 mmol/L	118-132 mmol/L
*Salmonella typhi* RT-PCR	Detected	-

**Figure 2:**
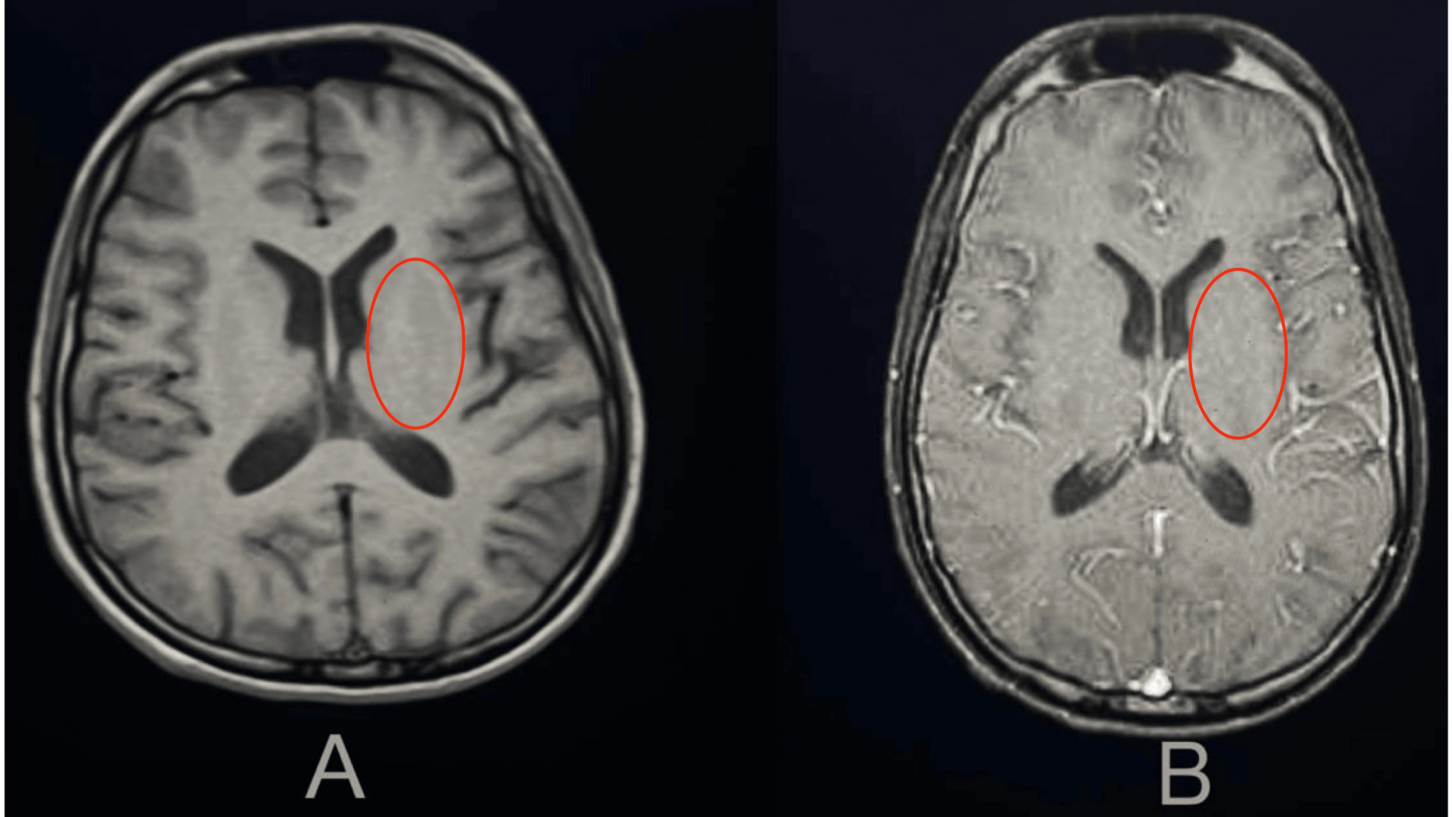
Brain MRI image cuts at the basal ganglia and caudate nucleus level A: Pre-contrast. B: Post-contrast. The red oval highlights the basal ganglia and caudate nucleus in both images. MRI: magnetic resonance imaging

Brain MRI with contrast was done to look for signs of encephalitis, which was a normal study (Figure 2).

Preliminary blood culture for enteric fever showed growth of *Salmonella typhi*, which was confirmed by IgM enzyme-linked immunosorbent assay (ELISA), and the patient was immediately started on injection ceftriaxone 2 g intravenously twice daily. CSF analysis came positive for *Salmonella typhi* through RT-PCR and negative for dengue virus. Platelet count monitoring was done every 24 hours, which was initially decreasing in trend with the lowest value being 72,000/cu mm. An electroencephalogram (EEG) was done, which showed a normal pattern. The patient had on and off movements for a total period of three days, with intensity depending on sedation level slowly decreasing in amplitude and frequency and at last to nil.

The patient was extubated, transferred to the ward, and discharged after four days with no abnormal movements at the time of discharge.

## Discussion

Dengue fever, one of the most notorious vector-borne diseases, is resurging around the world. In densely populated developing countries, the incidence of cases is rising exponentially daily. Therefore, it is thus utmost necessary to review its current epidemiology, its transmission, and the management options available. Dengue virus (DV) has positively stranded encapsulated RNA and belongs to the family Flaviviridae, having four serotypes referred to as DEN-1 to DEN-4 [5]. These serotypes are most commonly transmitted by mosquitoes *Aedes aegypti* and *Aedes albopictus* [6].

There have been several studies on pathogenesis and immune response in dengue virus infection. The primary immunological reaction triggered by a dengue virus infection is humoral. It stimulates the spleen of mice to produce helper T cells (ThF), which boosts the DV-specific B cells' clonal proliferation [7]. On the other hand, in DV-infected mice and humans, CD4+ T cells generate cytotoxic factor (CF), a distinct cytokine. In terms of structure, CF differs from all known proteins that have been analyzed in its amino-terminal sequence [8]. This cytotoxic factor is present in the sera of the majority of DV-infected persons, and in the most severe instances of dengue hemorrhagic fever, its values can be quite high [9].

Typhoid fever is an invasive bacterial infection seen in many parts of the world, predominantly in Asia and Africa. Typhoid is primarily seen in individuals with ages ranging from infancy to teenage. The culprit organism is *Salmonella enterica* (subspecies enterica serovar). It is passed via the fecal-oral route [10].

Choreoathetosis is usually a chronic movement disorder but can occur paroxysmally and disappear with correction of cause. This is called paroxysmal dystonic choreoathetosis. It is often misdiagnosed as a seizure episode. Primary paroxysmal dyskinesias are often hereditary. Ballism is considered an extreme kind of chorea, characterized by strong, tossing motions. It comes from the Greek word "ballismos," which means "jumping about or dancing." This is most commonly observed unilaterally or on one side of the body, but it can also occur bilaterally, in which case it is called biballism [11]. Secondary paroxysmal dyskinesias (PxD) have been previously reported in patients with conditions such as multiple sclerosis, head trauma, lacunar infarcts, migraine, and metabolic disorders such as hyperglycemia and hypocalcemia, as well as in central nervous system (CNS) infections. The causative lesions generally involve the medulla, basal ganglia structures, and rarely the spinal cord [12].

These infection-related movement disorders (IRMD) over the past few decades have been noted in many medical centers. However, these acute-onset movement disorders are generally secondary to acute insults in the body and often have a good prognosis once the root problem is corrected. The main cause of the above-seen movements in this case is secondary to typhoid encephalopathy along with a normal course of dengue virus infection.

The pathophysiology of this can be due to primary hyperactivity of neuronal tissues, loss of negative inhibitory drive, or channelopathies [13]. Understanding fundamental pathophysiology is crucial for effectively managing IRMD. There are several ways in which a disease-causing organism might trigger an immune response that leads to the development of autoimmunity. The first is molecular mimicry, an immunological occurrence in which epitopes found on pathogens can imitate or resemble a peptide sequence of host antigens. This can cause the activated host immunity to mistakenly target the host antigen, leading to an autoimmune response. Sydenham chorea exemplifies the phenomena in which antibodies targeting group A streptococcal polysaccharides exhibit cross-reactivity with human neural cells, leading to the development of chorea. The second process is epitope spreading, which refers to the gradual expansion of the initial immune response to an antigen to encompass new epitopes during inflammation. This can occur either through the recognition of new epitopes within the same antigen or through the presentation of previously "hidden" auto-antigens that are encountered during tissue injury. In addition to molecular mimicry, this pathway is also hypothesized to be involved in the development of anti-N-methyl-D-aspartate (NMDA) receptor encephalitis following herpes simplex encephalitis. The third method is bystander activation, where lymphocytes, particularly T cells, can be activated in an antigen-independent manner, leading to a cytokine storm. This activation occurs in addition to the immune response triggered by the extremely virulent disease. This can lead to the impairment of the blood-brain barrier and neuronal dysfunction, as shown in conditions such as postmalarial ataxia, dengue-related neurological syndromes, and more recently, COVID-19-related neurological syndromes. The fourth mechanism involves the stimulation of many B cells due to chronic infection, leading to the creation of autoantibodies. This process contributes to the development of autoimmunity, as observed in cases such as hepatitis C virus infection. In addition to the causative organism, the management of these infections also relies heavily on the mechanism by which they cause a certain movement disorder. Often, there might be many mechanisms at play within a single organism, a specific clinical presentation, or even an individual patient [14]. An effective and suitable management plan is crucial for enhancing positive long-term results.

## Conclusions

While dengue and typhoid are not uncommon infections, their occurrence together is rare. The clinical course with a fearsome sign makes this case a rare presentation and emphasizes that infections can be broad in their clinical manifestations and the need for open-mindedness while approaching a case. The occurrence of movement disorders is still completely unclear, and this case is an example that these can be seen in any instance.
